# Association of Visual Tracking Metrics With Post-concussion Symptomatology

**DOI:** 10.3389/fneur.2018.00611

**Published:** 2018-07-26

**Authors:** Jun Maruta, Lisa A. Spielman, Umesh Rajashekar, Jamshid Ghajar

**Affiliations:** ^1^Brain Trauma Foundation, New York, NY, United States; ^2^Department of Neurosurgery, Stanford University, Stanford, CA, United States; ^3^Department of Rehabilitation Medicine, Icahn School of Medicine at Mount Sinai, New York, NY, United States

**Keywords:** closed head injury, mild traumatic brain injury, ocular pursuit, predictive timing, smooth pursuit

## Abstract

Attention impairment may provide a cohesive neurobiological explanation for clusters of clinical symptoms that occur after a concussion; therefore, objective quantification of attention is needed. Visually tracking a moving target is an attention-dependent sensorimotor function, and eye movement can be recorded easily and objectively to quantify performance. Our previous work suggested the utility of gaze-target synchronization metrics of a predictive visual tracking task in concussion screening and recovery monitoring. Another objectively quantifiable performance measure frequently suggested for concussion screening is simple visuo-manual reaction time (simple reaction time, SRT). Here, we used visual tracking and SRT tasks to assess changes between pre- and within-2-week post-concussion performances and explore their relationships to post-concussion symptomatology. Athletes participating in organized competitive sports were recruited. Visual tracking and SRT records were collected from the recruited athlete pool as baseline measures over a 4-year period. When athletes experienced a concussion, they were re-assessed within 2 weeks of their injury. We present the data from a total of 29 concussed athletes. Post-concussion symptom burden was assessed with the Rivermead Post-Concussion Symptoms Questionnaire and subscales of the Brain Injury Screening Questionnaire. Post-concussion changes in visual tracking and SRT performance were examined using a paired *t*-test. Correlations of changes in visual tracking and SRT performance to symptom burden were examined using Pearson's coefficients. Post-concussion changes in visual tracking performance were not consistent among the athletes. However, changes in several visual tracking metrics had moderate to strong correlations to symptom scales (*r* up to 0.68). On the other hand, while post-concussion SRT performance was reduced (*p* < 0.01), the changes in the performance metrics were not meaningfully correlated to symptomatology (*r* ≤ 0.33). Results suggest that visual tracking performance metrics reflect clinical symptoms when assessed within 2 weeks of concussion. Evaluation of concussion requires assessments in multiple domains because the clinical profiles are heterogeneous. While most individuals show recovery within a week of injury, others experience prolonged recovery periods. Visual tracking performance metrics may serve as a biomarker of debilitating symptoms of concussion implicating attention as a root cause of such pathologies.

## Introduction

Cognitive abilities are often compromised after a concussion, with attention dysfunction being a particularly common sequela (1, 2). Since attention is a vital component of everyday functioning and even minor impairment may be subjectively debilitating, assessment of attention needs to be a key component of outcome measures for concussion. Furthermore, attention impairment may provide a cohesive neurobiological explanation for clusters of clinical symptoms that occur after a concussion (3–5). For these reasons, objective quantification of attention is needed. Given that eye-movement and attention processes share common neuronal circuitry (6, 7) and that eye movement can be recorded quickly and objectively to quantify performance, an outcome measure for post-concussion attentional functioning may be soundly derived from eye movement testing.

Visual tracking, also known as ocular pursuit, supports perceptual stability of a moving object of interest with a combination of saccadic and smooth pursuit eye movements. Visual tracking requires multiple components of attention, including target selection, sustained engagement, spatio-temporal working memory, prediction of the temporal course of the stimulus, and binding of the prediction and the required action (8–12). In particular, because the visual and motor processing delay of ≈100 ms (8, 13) would preclude visual interception of the target if one was to simply react to the visual input of the target, accurate and precise synchronization must be achieved through shaping anticipatory eye movement. Such shaping is likely to take place with contributions of the fronto-parietal cortical regions and the cerebellum (14), which are important foci of attention processing. Visual tracking performance is known to be, while variable between individuals, normally stable over time periods of weeks to months, and changes detected outside the limit of reliability express alterations in the brain (12, 15–17). Our previous work, using a predictive visual tracking task in combination with neuropsychological assessment and diffusion tensor imaging, associated visual tracking synchronization metrics with cognitive functioning and integrity of frontal white matter tracts that are vulnerable to a concussive impact (18). There is also vulnerability of the cerebellum to concussion (19–21). Therefore, a predictive visual tracking task would be a useful test paradigm to provide attentional bases for concussion screening and recovery monitoring with objective sensorimotor synchronization metrics (5, 18, 22).

Simple visuo-manual reaction time (simple reaction time, SRT) is another objectively quantifiable measure suggested for concussion screening (23, 24). An SRT task is also considered attention-dependent, invoking vigilance and a top-down control of detecting the target (23, 25). As with visual tracking, SRT performance shows both inter-individual variability and within-individual stability over a normal course of time (26–28), bearing biometric characteristics. However, the reactive nature of the behavior contrasts with predictive visual tracking, and the two behaviors may allude to the wellness of separate neural substrates (29).

Concussion clinical profiles are heterogeneous and may require subtyping to facilitate targeted treatments (30–32). While we have sought a unified explanation for divergent post-concussion clinical symptoms with attention impairment (4, 5), that attention too is multi-faceted and supported by sub-processes (33, 34) complicates matters. As yet, associations between clinical symptoms and cognitive impairments that follow a concussion are not clear (24, 35–37). There is indeed much to be explored beyond mere detection of concussion and toward characterization of heterogeneous traits. Here, we used visual tracking and SRT tasks to assess changes between pre- and within-2-week post-concussion performances and explore their relationships to post-concussion symptomatology.

## Materials and methods

### Subject enrollment

The subject enrollment and testing protocols were approved by the institutional review boards of Weill Cornell Medical College (WCMC) in New York, and Stanford University in California. In collaboration with school, university, and community athletic organizations in respective local areas, middle-school through adult athletes were enrolled and baseline tested. Prior to data collection, written informed consent by adult subjects, or legal guardians of minor subjects with the minors' assent, was obtained in accordance with the Declaration of Helsinki. Testing was conducted inside a parked recreational vehicle outfitted as a mobile testing site, or at the Citigroup Biomedical Imaging Center at WCMC. Data collection spanned from September of 2012 through September of 2016.

By research design, the athletes were retrospectively screened after the baseline testing for inclusion in the current analysis. Pre-screening for eligibility was deemed inappropriate because of the likelihood of singling out ineligible members from others belonging to same teams. The inclusion criteria were participation in organized competitive athletic activity, being of age 12–30, normal or corrected to normal vision, and for athletes over the age 18, a high school diploma or equivalent, or expected timely high school graduation. The exclusion criteria were a prior history of traumatic brain injury (including concussion), alcohol or substance abuse, a known neurologic disorder, or a psychiatric condition previously known or identified using questionnaires for attention deficit hyperactivity disorder, depression, and anxiety disorders, and a known vision-related disease or abnormality. Of a total of 3,091 athletes initially enrolled, 1,442 were selected for the study. More than one half of baselined sample (903 athletes) was excluded for a prior injury to the head.

During the baseline consent process, the athletes were given the option of allowing their athletic director, trainer, coach, or school nurse to contact the researchers if they sustain a concussion, as well as the research staff to contact the athletic staff to check on injury status. A diagnosis by a physician was not required. Prospective acute post-concussion enrollment was based on inclusion criteria consisting of an experience within 2 weeks of a concussion that resulted in loss of consciousness, post-traumatic amnesia, dizziness, nausea, headaches, balance problems, blurred or double vision, or daze and confusion, and on an exclusion criterion of intoxication at the time of injury.

### Visual tracking test

The details of the eye movement testing methods were described previously (12). Briefly, subjects performed a visual tracking task on a video-based eye tracker integrated with stimulus-presentation (EyeLink 1000, SR Research Ltd., Mississauga, Ontario, Canada). The stimulus was presented on a 120 Hz LCD monitor (SyncMaster 2233RZ, Samsung, Seoul, South Korea). The stimulus consisted of a red target of 0.5° of visual angle that was presented against a black background. The target traveled at a constant speed of 25.1°/s in a clockwise direction along a circular path with a radius of 10°. The task was performed in a normally lit room while subjects sat with their head stabilized by a chin-head rest. The semi-automated testing sequence lasted ~5 min, and included text and recorded audio instructions, a 5-s practice run, calibration, validation, and two 15-s test runs. The visual acuity of each subject was confirmed to be normal or corrected-to-normal prior to testing using a handheld vision chart.

Collected eye movement data were screened with an automated algorithm for >10% of missing data, artifacts associated with inadequate quality of calibration, or poor head stabilization during recording as evidenced by a large change in visual fixation records and were also visually inspected. Validated eye movement data were processed using a standardized method to derive performance metrics. To characterize the stability of the gaze on the target, we evaluated the variability of gaze position error along axes orthogonal (radial) and parallel (tangential) to target movement (standard deviation of radial and tangential errors—SDRE, SDTE). The smaller the SDRE or SDTE value, the more precise the tracking. To characterize the central tendency of gaze position relative to the target, we evaluated the mean radial error and the mean phase error. A negative radial error indicated the gaze drawing a smaller circular trajectory than the target. A negative phase error indicated the gaze trailing the target or phase lag. We also computed the horizontal and vertical smooth pursuit velocity gains (H and V gains), which were the ratios between the amplitudes of modulation of smooth pursuit eye velocity and target velocity. A smaller gain indicated less accurate tracking.

### Simple reaction time test

We collected simple visuo-manual reaction time data using the SRT component of the Automated Neuropsychological Assessment Metrics Version 4 (ANAM4) (38). The SRT test was added to the testing protocol after the initiation of the study; therefore subjects who were enrolled in the study at an early stage did not receive this test. Other components of the ANAM4 library were not deployed. The stimulus was a large asterisk symbol presented at the center of a blank computer screen. The subjects were instructed to press a response key as quickly as possible each time the stimulus was presented. There were 40 trials in a test and the results were automatically analyzed by the software. The software discarded a response made in <130 ms after the cue presentation in analysis. We chose the median and throughput outputs to characterize the subjects' performance. Median was used to describe each subject's central tendency of reaction times, the intra-individual distribution of which is typically skewed. The throughput metric was defined as the number of proper responses per min.

### Symptom assessment

The Rivermead Post-Concussion Symptoms Questionnaire (RPQ) (39) measures the severity of 16 problems that commonly follow a concussion. The subjects were asked to rate changes in symptoms from 0 to 4 in comparison to the pre-injury state, corresponding to “not experienced,” “no more of a problem,” “mild problem,” “moderate problem,” and “severe problem,” and total scores were calculated as the sum of all symptom scores with a range from 0 to 64. Types of symptoms were not analyzed based on sub-test scores of the RPQ (40).

The Brain Injury Screening Questionnaire (BISQ) (41) is a screening tool for documenting lifetime history of traumatic brain injury and the presence of current symptoms and for ruling out alternative explanations for reported symptoms. A section of BISQ is an inventory of 100 four-point-rated symptoms that characterize a comprehensive range of transient and chronic problems after brain injury. A factor analysis of these symptoms revealed four main factors (42), which were labeled as memory-attention, depression-anxiety-mood, aggression-impulsivity, and physical. Symptom factor scores are scaled from 0 to 3, with 0 indicating no symptoms.

### Statistical analysis

We analyzed changes in visual tracking and SRT performances and their relationship to post-concussion symptomatology. Within-individual changes in visual tracking and SRT performances before and after injury were tested with paired *t*-tests. Relationships between visual tracking and SRT performances and those between changes in performance and post-concussion symptoms were examined with Pearson's correlation coefficient. Analyses were based on all observed data without case deletion or replacing missing data with substituted values. Findings are presented with a statistical significance defined by the alpha level of 0.05, and without correction for multiple comparisons because of the exploratory nature of the study.

## Results

In the baseline-tested 1,442 athletes selected for the study, the research staff was made aware of and was able to test 29 cases of concussion meeting the enrollment criteria. There were 14 female and 15 male subjects. The subjects' ages spanned from 14 years and 2 months to 22 years and 5 months, with a mean (SD) of 18.4 (2.3) years. The post-concussion testing took place with the mean (SD) of 5.3 (3.3) days after the injury, and 2.8 (2.5) months after the baseline testing. Valid visual tracking scores from both baseline and acute post-injury assessments were available from 20 of the 29 subjects. Most of the eye tracking technical failures were associated with poor head stabilization. Four of the 29 subjects did not undergo the SRT test at baseline as they were enrolled before the test was implemented in the testing protocol. A maximum of five anticipatory responses and no timeouts were recorded in any subject. Most tests were completed with 40 valid trials with a mean (SD) of 39.4 (1.0) trials. Altogether, valid baseline and acute post-injury SRT scores were available from 25 subjects. All 29 subjects had RPQ and BISQ scores.

**Figure 1:**
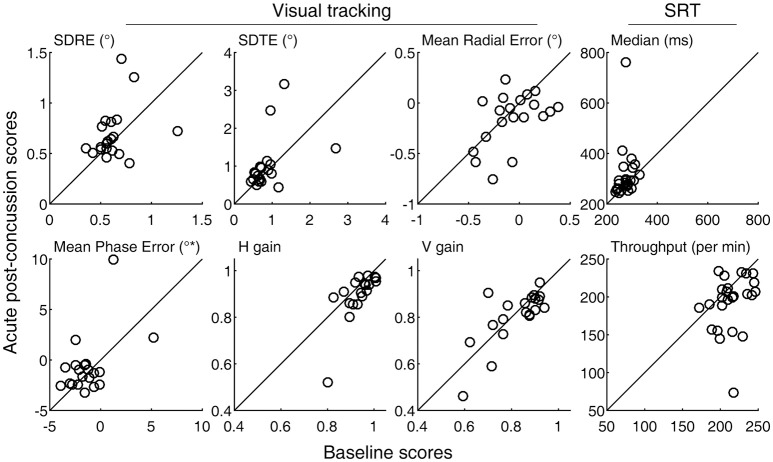
Baseline and acute post-concussion performance scores. Circle markers in each panel indicate individual subjects. The diagonal lines indicate no change from the baseline measurement. Also see Table 1.

**Figure 2:**
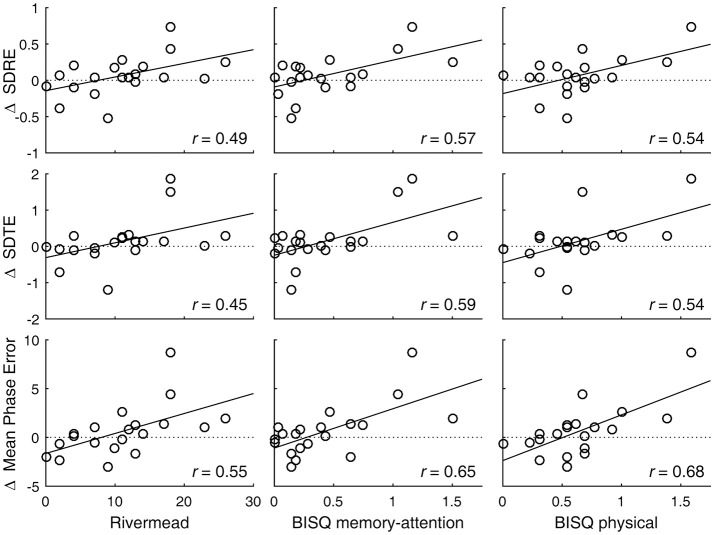
Visual tracking performance changes are associated with post-concussion symptomatology. Circle markers in each panel indicate individual subjects. A regression line is drawn to indicate a trend. A positive change in SDRE and SDTE indicates an increased gaze position variability during tracking of a moving target. A positive change in mean phase error indicates a phase advancement. Also see Table 2.

A degradation in visual tracking performance after experiencing a concussion was not observed consistently, with only H gain showing a change at *p* < 0.05 (Table 1; Figure 1). However, changes in SDRE, SDTE, and mean phase error moderately correlated with RPQ scores, with increased gaze position variability and a phase lead being associated with reports of more severe symptomatology that presented after a concussion (Table 2; Figure 2, leftmost column). The RPQ scores ranged from 0 to 26 (mean 12.2) among the 29 subjects, and from 0 to 26 (mean 11.1) among the 20 subjects with valid baseline and post-injury visual tracking scores. The same visual tracking metrics also correlated with the memory-attention and physical symptom subscale scores of the BISQ at a moderate to strong level (Figure 2, middle and rightmost columns). Table 3 shows a sample of questionnaire items factorized together under these subscales. Correlations between visual tracking metrics and the BISQ depression-anxiety-mood and aggression-impulsivity symptom subscales were weak at best and not statistically significant (Table 2).

**Table 1:** Within-individual changes from the baseline in visual tracking and reaction time performances within 2 weeks after a concussion.

	**Baseline**	**Post-concussion**	**Mean change**	**|*t*|**	***P*-value**
**VISUAL TRACKING (*****N*** = **20)**					
SDRE (°)	0.622 (0.180)	0.688 (0.252)	0.065	1.08	0.29
SDTE (°)	0.857 (0.472)	0.998 (0.661)	0.141	0.99	0.34
Mean radial error (°)	−0.068 (0.231)	−0.155 (0.256)	−0.087	1.51	0.15
Mean phase error (°^*^)	−1.323 (1.924)	−0.705 (2.800)	0.618	1.06	0.30
H gain	0.936 (0.055)	0.898 (0.098)	−**0.038**	**2.44**	**0.025**
V gain	0.821 (0.102)	0.806 (0.114)	−0.014	0.80	0.43
**SRT (*****N*** = **25)**					
Median (ms)	275.4 (24.0)	314.1 (100.5)	38.6	1.91	0.068
Throughput (per min)	214.0 (19.5)	192.4 (35.8)	−**21.6**	**2.90**	**0.008**

Baseline and post-concussion entries indicate group mean (SD). Bold typeface indicates p < 0.05. SDRE, SDTE, and mean radial error are expressed in degrees of visual angle (°) while mean phase error is in degrees of phase angle (°^*^). H and V gains are dimensionless quantities.

**Table 2:** Pearson correlations between performance changes and symptom scores.

	**RPQ**	**BISQ**			
	**Total**	**Memory-attention**	**Depression-anxiety-mood**	**Aggression-impulsivity**	**Physical**
**VISUAL TRACKING (*****N*** = **20)**					
SDRE	0.49^*^	0.57^**^	0.10	0.06	0.54^*^
SDTE	0.45^*^	0.59^**^	0.16	0.13	0.54^*^
Mean radial error	0.06	−0.34	−0.07	−0.13	−0.26
Mean phase error	0.55^*^	0.65^**^	0.27	0.35	0.68^**^
H gain	−0.11	−0.26	0.06	−0.05	−0.42
V gain	−0.17	−0.07	0.11	0.35	−0.23
**SRT (*****N*** = **25)**					
Median	−0.02	−0.16	−0.33	−0.27	−0.20
Throughput	−0.14	−0.01	0.20	0.12	0.13

Bold typeface indicates statistical significance,

* p < 0.05,

** *p < 0.01*.

**Table 3:** Example symptom items factorized together under memory-attention and physical subscales of BISQ.

**Memory and attention items**	**Physical symptom items**
Becoming confused in familiar places Being easily distracted Forgetting what happened yesterday Forgetting names of people Forgetting to turn off appliances Difficulty making decisions Difficulty solving problems Difficulty planning future events Difficulty following instructions, written or oral Difficulty learning new skills and information Forgetting what you just read Difficulties with reading, writing and math Difficulty making conversation	Having trouble staying awake Having trouble falling asleep or staying asleep Having trouble waking up after sleep or a nap Feeling cold Feeling dizzy Experiencing ringing in the ears or having trouble hearing Having double vision or blurred vision Food not tasting right Having headaches Increased or decreased sexual interest or behavior Avoiding family members or friends

**Figure 3:**
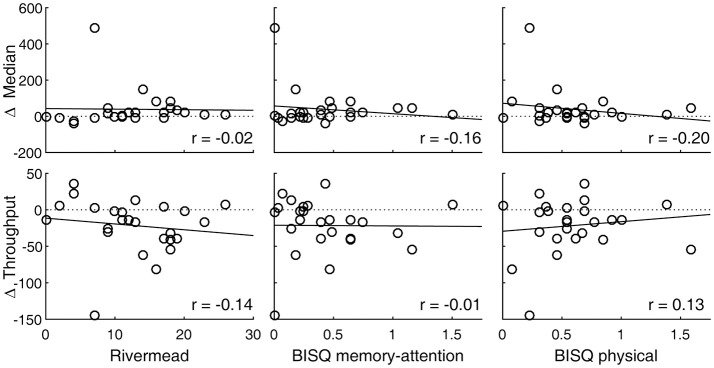
SRT performance changes are associated with post-concussion symptomatology. Circle markers in each panel indicate individual subjects. A regression line is drawn to indicate a trend. A positive change in median or a negative change in throughput indicates slowed reaction. Also see Table 2.

SRT performance was degraded after experiencing a concussion, with the throughput metric showing a statistically significant change (*p* = 0.008, Table 1). Measured in terms of median, reaction times were also longer overall, although a *t*-test failed to show a statistically significant change because one subject representing an extreme, rather than a reverse, of the trend contributed to the overall increase in the sample variance (Figure 1, top right panel). An inspection of this subject's raw reaction time records showed 40 valid responses ranging from 434 to 1,404 ms. While this subject also had the lowest throughput after the concussion, with the throughput metric being reciprocally related to the mean of reaction times, the deviation associated with slowed response was less exaggerated. The visual tracking performance of this subject was not exceptional either at baseline or post-injury, or in terms of changes between baseline and post-injury scores. The SRT metrics did not meaningfully correlate with any of the symptom scores (Table 2; Figure 3).

Normal interdependence between visual tracking and SRT metrics is low (29), but changes in the overall integrity of the attention system may impact across its different components (18, 43). We thus tested for correlations between visual tracking and SRT metrics in terms of their post-concussion changes from the baseline. However, among the 19 subjects who had valid baseline and post-concussion data for both visual tracking and SRT tests, the correlation between changes were very weak at best (*r* < 0.20) and not statistically significant.

## Discussion

The normally expected within-individual stability of visual tracking performance can be disrupted by a concussion. We found that when baseline-tested athlete subjects were reassessed within 2 weeks of a concussion, changes in visual tracking performance reflected clinical symptoms. Post-concussion symptom burden was assessed with the RPQ, which evaluates differences from the pre-injury state, and with the BISQ, which quantifies symptom severity in four separate dimensions. Symptomatic subjects had reduced precision and accuracy of gaze-target synchronization. Specifically, these visual tracking performance changes were linked to memory-attention and physical symptoms, but not with depression-anxiety-mood or aggression-impulsivity symptoms.

Visuo-manual reaction was slowed following a concussion. This finding substantiates that the impact to the head sustained by the athlete subjects in fact had a neurological effect. This affirmation is despite the fact that the testing usually took place days after the injury, but is consistent with a previous report that, even though the greatest recovery from clinical symptoms typically takes place during the first several days after the injury (44, 45), a reaction deficit can persist for a longer period (35). Such apparent dissociation between degraded reaction performance and the contemporaneous subjective symptomatology is consistent with the results of the direct correlation analysis that we conducted. It is not clear whether slowed response becomes apparent to the individual in a subtle way or whether it is coupled to symptoms that we did not quantify. It is also not clear how attention subcomponents that support visual tracking and SRT performances interact in daily activities under normal and post-concussion conditions.

Because of heterogeneity in the presentations of concussion and the possible existence of clinical subtypes (30–32), evaluation of concussion requires assessments in multiple domains. As stated in INTRODUCTION, predictive visual tracking and reaction time measurements may allude to the wellness of separate neural substrates (29). The current results with visual tracking and reaction time metrics reflecting different aspects of post-concussion profiles support this view. In particular, we have postulated that impaired predictive timing, as measured by visual tracking synchronization metrics, is the primary basis for the post-concussion attention deficit and that further cognitive, somatic, or affective symptoms can be explained as secondary consequences of impaired predictive timing (3–5). That visual tracking metrics were linked to memory-attention and physical symptoms are in line with these accounts, and these metrics may serve as an attentional biomarker that quantifies the impact associated with debilitating symptoms within these domains. On the other hand, symptoms in depression-anxiety-mood and aggression-impulsivity domains require alternative explanations because similar links were not found. Assessment of impairments to other sensorimotor systems such as proprioceptive, vestibular, and ocular vergence mechanisms should present further clues to the puzzle (32, 46, 47).

The sample was a prospective cohort from a natural setting of competitive sports. Although an enormous amount of resources was devoted for baseline testing, the culminated sample size was limited. Over one half of the baselined athletes met the exclusion criteria, the primary reason being a prior head injury. Consequently, our sample represents only a selective subgroup of a typical athlete population. The sample size was unfortunately further reduced because the timing of data processing was determined by the prospective study design and we lacked the means to screen for the validity of eye movement records at the time of data collection. Improvements in eye tracking technology supplemented by immediately screening the data with an automated algorithm should reduce the technical failure rate. Also constrained by the study design was the timing of post-concussion assessment, which was variable within the 2-week window. Since trajectories of symptom recovery vary among individuals (35, 37), it was unclear in the present study if individuals with low symptom scores were those who recovered or those who were asymptomatic. A study with an initial assessment timed closer to the injury and with longitudinal follow-ups would inform varying trajectories of recovery following a concussion.

## Conclusion

Post-concussion symptoms are subjective but can be debilitating. Evaluation of concussion requires assessments in multiple domains because the clinical profiles are heterogeneous and patients can benefit from targeted treatments. However, it is difficult to determine the root causes of various clinical symptoms. While most concussed individuals are said to subjectively experience recovery within a week of injury, deficits in objective measures may linger for some. Still others experience prolonged recovery periods. We sought to associate objective attention measures with clinical symptoms to elucidate their underlying pathophysiology. Quantification of attention, particularly predictive timing, based on visual tracking performance may render a biological explanation for cognitive and physical symptoms that occur after a concussion as a result of changes in the supporting neural circuit.

## Author contributions

JM and JG designed experiments and oversaw data collection and analysis. JM, LS, and UR contributed to data management and conducted the statistical analyses. JM drafted the manuscript. All authors contributed to the interpretation of data and to revising the work.

### Conflict of interest statement

JG is director of Sync-Think, Inc. and the inventor of U.S. patent 7,384,399. JM holds stock option in Sync-Think. LS and UR have a consulting arrangement with Sync-Think. JM, UR, and JG are inventors of pending patent application PCT/US2016/027923 related to the subject matter described in this article. The authors declare no other potential conflicts of interest with respect to the research, authorship, and/or publication of this article.

## Abbreviations

BISQ: Brain Injury Screening Questionnaire
RPQ: Rivermead Post-Concussion Symptoms Questionnaire
SD: standard deviation
SDRE: SD of radial errors
SDTE: SD of tangential errors
SRT: simple reaction time.
